# Supplementary material for: Coordination and variability of muscular activation in male athletes with and without subacromial impingement syndrome: A case-control study
Source: PLoS One. 2025 Feb 26;20(2):e0319048. doi: 10.1371/journal.pone.0319048 (PMC11864556; doi:10.1371/journal.pone.0319048)
Supplement: Attachment — Submitted filename: Review PONE-D-24-52678.pdf [file pone.0319048.s002.pdf]

Dear Authors,

Special thanks for your manuscript submission and to the editors for providing the opportunity to review this work.

The study, titled **Coordination and Variability of Muscular Activation in Male Athletes with and Without Subacromial Impingement Syndrome: A Case-Control Study**,' aims to investigate and compare muscular coordination and variability during repetitive shoulder movements among athletes with and without SIS.

The manuscript is engaging, and here are some insights and suggestions:

- This research has the potential to interest the **PLOS ONE** readership but requires significant improvement.
- The abstract is unclear. Enhance it by clearly stating the study's aim, methodology, and conclusions for better clarity and comprehension.
- The authors investigate "the coordination and variability of muscular activation" using NNMF to extract muscle synergies. Additionally, since simultaneous activation around the shoulder joint can provide valuable insights into joint stabilization and movement precision, consider incorporating muscle co-activation analysis into the study.
- Use a single method (either tables or figures) to present results; avoid redundant representations, such as the overlap between Table 2 and Figure 3, or Table 3 and Figure 4.
- Revise the placement of Figure 6 and its legend to improve readability and alignment with the text.
- While the paper is well-illustrated and presented, it should be significantly reduced in length to focus on content directly relevant to the study's aim.
- The study's limitations are only briefly mentioned in the abstract. It is preferable to introduce a separate section for limitations and future work before the conclusions section.
- Although the conclusions section addresses the study's aim, it is too general. Improve it by incorporating the key findings and important results from the study.
