# Supplementary material for: Coordination and variability of muscular activation in male athletes with and without subacromial impingement syndrome: A case-control study
Source: PLoS One. 2025 Feb 26;20(2):e0319048. doi: 10.1371/journal.pone.0319048 (PMC11864556; doi:10.1371/journal.pone.0319048)
Supplement: Attachment — Submitted filename: Point by point response to reviewers.docx [file pone.0319048.s003.docx]

**Comments to the Author**

1. Is the manuscript technically sound, and do the data support the conclusions?

The manuscript must describe a technically sound piece of scientific research with data that supports the conclusions. Experiments must have been conducted rigorously, with appropriate controls, replication, and sample sizes. The conclusions must be drawn appropriately based on the data presented.

Reviewer #1: Yes

Reviewer #2: Yes

Thank you for considering our work.

2. Has the statistical analysis been performed appropriately and rigorously?

Reviewer #1: Yes

Reviewer #2: Yes

Thank you for considering our work.

3. Have the authors made all data underlying the findings in their manuscript fully available?

The [PLOS Data policy](http://track.editorialmanager.com/CL0/http:%2F%2Fwww.plosone.org%2Fstatic%2Fpolicies.action%23sharing/1/010f0194277e64ad-d100b73c-cd58-4388-861c-c2e5f69fd2e5-000000/zHWvsFwKOPbR3QkG4a19Pfv4vqmqus2svk6BQtLPQ1Y=191) requires authors to make all data underlying the findings described in their manuscript fully available without restriction, with rare exception (please refer to the Data Availability Statement in the manuscript PDF file). The data should be provided as part of the manuscript or its supporting information, or deposited to a public repository. For example, in addition to summary statistics, the data points behind means, medians and variance measures should be available. If there are restrictions on publicly sharing data—e.g. participant privacy or use of data from a third party—those must be specified.

Reviewer #1: Yes

Reviewer #2: Yes

Thank you for considering our work.

4. Is the manuscript presented in an intelligible fashion and written in standard English?

PLOS ONE does not copyedit accepted manuscripts, so the language in submitted articles must be clear, correct, and unambiguous. Any typographical or grammatical errors should be corrected at revision, so please note any specific errors here.

Reviewer #1: Yes

Reviewer #2: Yes

Thank you for considering our work.

5. Review Comments to the Author

Reviewer #1: Dear Authors,
Special thanks for your manuscript submission and to the editors for providing the opportunity to review this work.
The study, titled Coordination and Variability of Muscular Activation in Male Athletes with and Without Subacromial Impingement Syndrome: A Case-Control Study,' aims to investigate and compare muscular coordination and variability during repetitive shoulder movements among athletes with and without SIS.
The manuscript is engaging, and here are some insights and suggestions:
- This research has the potential to interest the PLOS ONE readership but requires significant improvement.
- The abstract is unclear. Enhance it by clearly stating the study's aim, methodology, and conclusions for better clarity and comprehension.

Thank you for your valuable comment. All parts were amended and highlighted.

- The authors investigate “the coordination and variability of muscular activation” using NNMF to extract muscle synergies. Additionally, since simultaneous activation around the shoulder joint can provide valuable insights into joint stabilization and movement precision, consider incorporating muscle co-activation analysis into the study.

Given that this study was not one of the primary objectives of this research and that the number of variables examined in this study is relatively large, this issue has not been examined. However, we have added this valuable point in the research limitations section.

- Use a single method (either tables or figures) to present results; avoid redundant representations, such as the overlap between Table 2 and Figure 3, or Table 3 and Figure 4.

Thank you for your valuable comment. Duplicates have been removed and necessary changes have been made to the text.

- Revise the placement of Figure 6 and its legend to improve readability and alignment with the text.

Thank you for your valuable comment. It is amended.

- While the paper is well-illustrated and presented, it should be significantly reduced in length to focus on content directly relevant to the study's aim.

Thank you for your valuable comment. Two figures were removed to shorten the text, and the authors also tried to summarize the text as much as possible. The word count of the article is based on the journal's standards. However, the large number of variables and the volume of results have increased the length of the article.

- The study's limitations are only briefly mentioned in the abstract. It is preferable to introduce a separate section for limitations and future work before the conclusions section.

Thank you for your valuable comment. The limitation section has been added to the text.

- Although the conclusions section addresses the study's aim, it is too general. Improve it by incorporating the key findings and important results from the study.

Thank you for your valuable comment. The conclusion changed.

Reviewer #2:

1) It is a interesting study however the authors need to elaborate why they have choosen the muscle synergies measurement and notmuscle activity?

Thank you for your valuable comment. We tried to add some text to introduction to address this issue.

2) what advatage it offers over the muscle acitvity?

Thank you for your valuable comment. We tried to add some text to introduction to address this issue.

3)the authors are advised to add comparision with muscle acitvities in SIS vide refercne "Sharma, S., Hussain, M. E., & Sharma, S. (2021). Effects of exercise therapy plus manual therapy on muscle activity, latency timing and SPADI score in shoulder impingement syndrome. Complementary Therapies in Clinical Practice, 44, 101390."

Thank you for your valuable comment. Added as reference.

Phadke, V., & Ludewig, P. M. (2013). Study of the scapular muscle latency and deactivation time in people with and without shoulder impingement. Journal of Electromyography and Kinesiology, 23(2), 469-475.

Thank you for your valuable comment. Added as reference.

4) the authors are requested to how weill the clinical physiotherpist use their findings to obtain good outcome

Thank you for your valuable comment. It is added to the discussion section, a paragraph before the limitations.
